# Factors influencing hospitalization or emergency department visits and mortality in type 2 diabetes following the onset of new cardiovascular diagnoses in a population-based study

**DOI:** 10.1186/s12933-024-02211-4

**Published:** 2024-04-10

**Authors:** Björn Agvall, Junmei Miao Jonasson, Alexander Galozy, Anders Halling

**Affiliations:** 1 Department of Research and Development, Region Halland, Halmstad, Sweden; 2 Center for Primary Health Care Research, Department of Clinical Sciences, Lund University, Malmö, Malmö, 202 13 Sweden; 3 School of Public Health and Community Medicine, University of Gothenburg, Göteborg, Sweden; 4 Center for Applied Intelligent Systems Research, Halmstad University, Halmstad, Sweden

**Keywords:** Type 2 diabetes, Cardiovascular disease, Healthcare utilization, Mortality

## Abstract

**Background:**

Individuals with type 2 diabetes (T2D) are at increased risk of developing cardiovascular disease (CVD) which necessitates monitoring of risk factors and appropriate pharmacotherapy. This study aimed to identify factors predicting emergency department visits, hospitalizations, and mortality among T2D patients after being newly diagnosed with CVD.

**Methods:**

In a retrospective observational study conducted in Region Halland, individuals aged > 40 years with T2D diagnosed between 2011 and 2019, and a new diagnosis of CVD between 2016 and 2019, were followed for one year from the date of CVD diagnosis. The first encounter for CVD diagnosis was categorized as inpatient-, outpatient-, primary-, or emergency department care. Follow-up included laboratory tests, blood pressure, pharmacotherapies, and healthcare utilization. Hazard ratios (HR) in two Cox regression analyses determined relative risks for emergency visits/hospitalization and mortality, adjusting for age, sex, glucose regulation, lipid levels, kidney function, blood pressure, pharmacotherapy, and healthcare utilization.

**Results:**

The study included a total of 1759 T2D individuals who received a new CVD diagnosis, with 67% diagnosed during inpatient care. The average hospitalization stay was 6.5 days, and primary care follow-up averaged 10.1 visits. Patients with CVD diagnosed in primary care had a HR 0.52 (confidence interval [CI] 0.35–0.77) for emergency department visits/hospitalization, but age had a HR 1.02 (CI 1.00-1.03). Pharmacotherapy with insulin, DPP4-inhibitors, aldosterone antagonists, and beta-blockers had a raised HR. Highest mortality risk was observed when CVD was diagnosed inpatient care, systolic blood pressure < 100 mm Hg and elevated HbA1c. Age had a HR 1.05 (CI 1.03–1.08), eGFR < 30 ml/min HR 1.46 (CI 1.01–2.11), and LDL-Cholesterol > 2,5 h 1.46 (CI 1.01–2.11) and associated with increased mortality risk. Pharmacotherapy with metformin had a HR 0.41 (CI 0.28–0.62), statins a HR 0.39 (CI 0.27–0.57), and a primary care follow-up < 30 days a HR 0.53 (CI 0.37–0.77) and associated with lower mortality risk.

**Conclusions:**

T2D individuals who had a new diagnosis of CVD were predominantly diagnosed when hospitalized, while follow-up typically occurred in primary care. Identifying factors that predict risks of mortality and hospitalization should be a focus of follow-up care, underscoring the critical role of primary care in the effective management of T2D and CVD.

**Supplementary Information:**

The online version contains supplementary material available at 10.1186/s12933-024-02211-4.

## Introduction

Diabetes affects roughly 537 million individuals worldwide, equating to one in every 11 adults [1, 2]. Diabetes is a complex condition, and its incidence is steadily increasing. In Sweden, it is estimated that approximately 5% of the population is affected [3]. Type 2 diabetes (T2D) is the most prevalent form, representing 90–95% of all cases. This condition predominantly afflicts adults and the elderly, with strong associations with genetic factors, obesity, and unhealthy behaviors like smoking, high alcohol consumption, and physical inactivity [4].

Diabetes mellitus, in general, has a two-fold excess risk of cardiovascular outcomes (coronary heart disease, ischemic stroke, and vascular deaths), independent of other risk factors [5, 6]. Individuals with a prolonged history of diabetes mellitus for an extended period and who exhibit microvascular complications, such as renal complications, will experience elevated relative risk levels for cardiovascular disease (CVD) [7]. A systematic review spanning the years 2007–2017 reported that, in total, 32% of all individuals with T2D also concurrently suffer from CVD [8]. The occurrence of CVD in T2D individuals is considered the leading cause of adverse health outcomes, e.g., hospitalization and mortality [8]. In a comprehensive study focused on T2D, the mortality rate caused by CVD was found to be around 5 per 1000 person-years in individuals with T2D compared with individuals without T2D. Within this cohort, the primary factors influencing outcomes were identified as the age at which diabetes was diagnosed, the degree of glycemic control, and the presence of renal complications [7]. The risk of CVD and coronary heart disease increases in individuals with T2D even when their HbA1c levels are < 53 mmol/mol and continue to increase with further increased glucose levels [7]. Individuals with T2D and an established history of CVD are classified as having an extremely high risk of new cardiovascular-related events [9].

To prevent the onset of CVD in individuals with T2D, it is essential to implement lifestyle changes, which encompass improvements in diet, increased physical activity, weight management, and quitting smoking [10]. Previous research has demonstrated the significance of lowering glucose levels, as a 1% reduction in glucose levels has been linked to a 15% decrease in the relative risk of CVD events, hospitalizations, and mortality [11, 12]. Similarly, managing blood pressure is of utmost importance, given that hypertension is associated with an elevated risk of both morbidity and mortality [13, 14]. Furthermore, a reduction in cholesterol levels induced by statin medication has also been correlated with a decreased risk of CVD [15].

Individuals diagnosed with T2D have an elevated risk of CVD thus making it crucial to closely monitor glucose, cholesterol, blood pressure levels and renal function and the follow-up of these individuals regarding medication. Still, it remains uncertain whether the management of these risk factors is efficient, where it is pursued and if patients are prescribed recommended treatments after being diagnosed with a new cardiovascular event. Furthermore, frequent communication lapses and insufficient information during the transition from hospital to primary care can adversely impact patient care [16, 17]. To the best of our knowledge, there is a shortage of research on factors that could be associated with emergency department visits, hospital admissions, and mortality among individuals with type 2 diabetes following a cardiovascular disease event. The objective was to identify the key predictors of emergency department visits, hospitalizations, and mortality among T2D patients after developing CVD.

## Method

This study was a retrospective, non-interventional observational study conducted within Region Halland, which is situated in the southwestern region of Sweden with a population of 340,000 residents. The healthcare infrastructure within this region is comprised of three acute care hospitals, 40 inpatient wards, two emergency departments, 30 outpatient specialty clinics and 46 primary care clinics. The study included 23 primary care clinics operating under private administration alongside an equal number under public administration.

### Data source

Region Halland possesses comprehensive access to pseudo-anonymized data through the Regional Healthcare Information Platform (RHIP) [18]. This dataset includes clinical, operational capacity, and financial information pertaining to all individuals who have received treatment since 2011 across all publicly-operated healthcare facilities within Region Halland. RHIP was the primary data source for this study, and a similar methodology has been previously used to investigate the population with heart failure within Region Halland [19, 20]. The data from RHIP includes primary care, emergency department care, hospital admissions and hospital outpatient care as well as inpatient care. It includes the complete Region Halland patient population linking clinical, operational, and cost information at the patient encounter level, together with system resource and capacity data (e.g., full-time equivalent nurses/physicians; hospital bed occupancy). RHIP also contains data concerning deceased patients including the date of death and therefore contains sufficient data to analyze all-cause mortality in the cohort. Information on dispensed drug treatment was retrieved from the National Drug Registry.

### Study population

The study included individuals aged ≥ 40 years who’d had a T2D diagnosis between 2009 and 2019 and received a new cardiovascular diagnosis sometime between 2016 and 2019 upon inclusion to this study. Patients who had a CVD diagnosis prior to 2016 were excluded. ICD-codes for cardiovascular diagnosis are displayed in Supplementary Table 1. The index date for each patient was when they were first diagnosed with a new cardiovascular diagnosis. All participants were living in Region Halland during the study period.

### Study process and measurements

The follow-up duration extended for one year from the index date, or until the patient’s death if that occurred earlier. The study procedure is displayed in Supplementary Fig. 1. Each patient was enrolled at the onset of their new cardiovascular diagnosis and concurrently when the new cardiovascular diagnosis was documented. The point at which the patient received the diagnosis was categorized into the following settings: hospital inpatient and outpatient care, primary care-, and the emergency department. Visits to the emergency department, outpatient care or primary care that led to a hospital admission within 48 h were defined as hospital inpatient care. The diagnosis defining the CVD and T2D is presented in Supplementary Table 1.

**Fig. 1:**
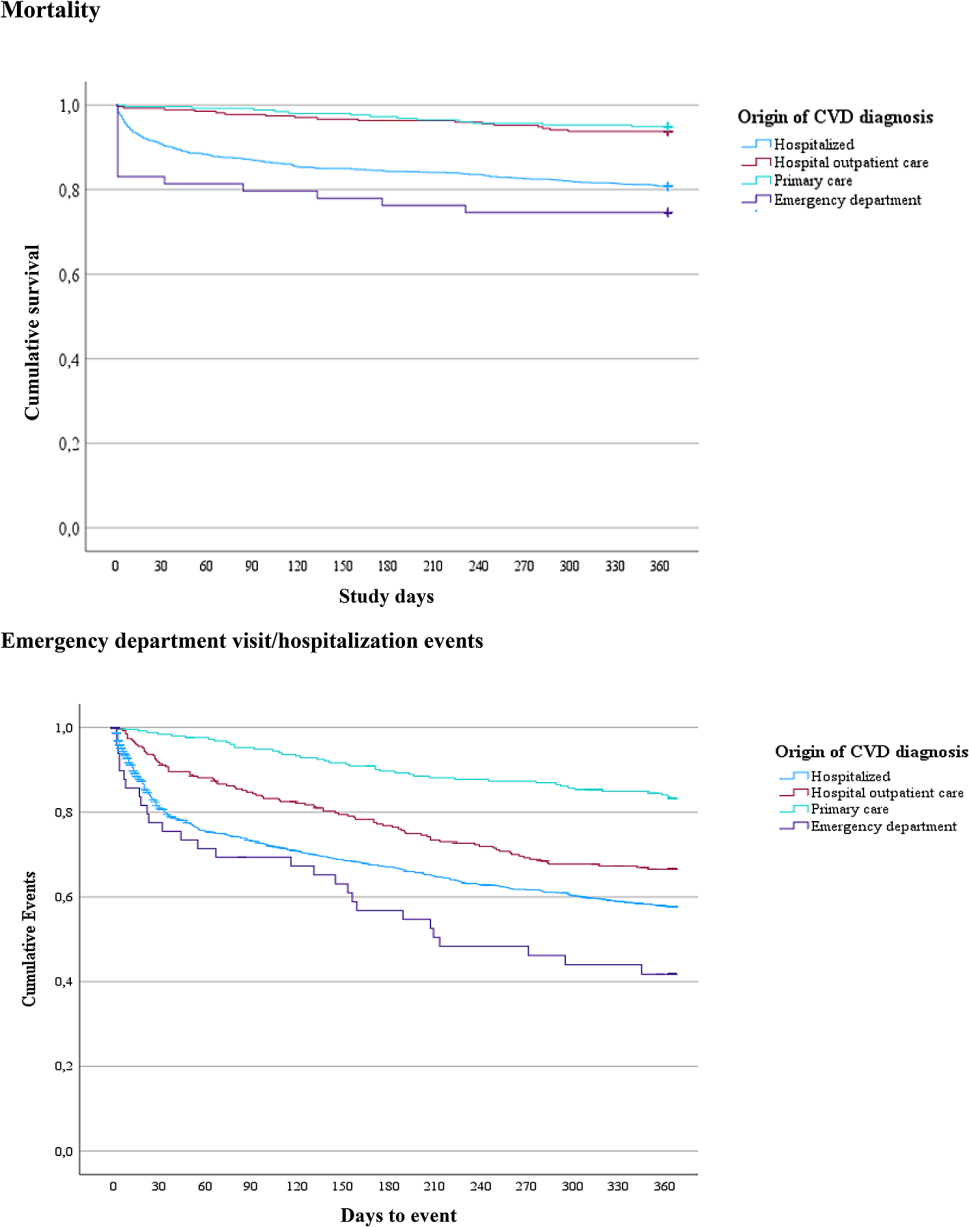
A Kaplan-Meier analysis depicting trends over time for the mortality and the events of emergency department visit/hospitalization, based and the mortality and events patterns over time for in-patient care, out-patient care, primary care, and the emergency department

Gathered at index were age at the index date, sex, comorbidities, specific cardiovascular diagnoses, estimated glomerular filtration rate (eGFR), glycated hemoglobin (HbA1c) levels, cholesterol values, and recorded blood pressure measurements. The specific diagnoses for comorbidities and cardiovascular diagnosis are listed in Supplementary Table 1. All laboratory results and blood pressure readings were collected over the entire study period and then averaged. The follow-up of laboratory values was those within three months before the study period ended. HbA1c values were categorized into four groups: <52 mmol/mol, 52–57 mmol/mol, 58–70 mmol/mol, and > 70 mmol/mol. Total cholesterol was classified as either ≥ 4.5 mmol/L or < 4.5 mmol/L, while LDL cholesterol was categorized as either ≥ 2.5 mmol/L or < 2.5 mmol/L. Renal function was stratified into three eGFR levels: >60 mL/min/1.73 m², 30–60 mL/min/1.73 m², and < 30 mL/min/1.73 m² [21, 22]. The urine albumin-to-creatinine ratio (UACR) was grouped into three categories: normal albuminuria (< 3 mg/mmol), microalbuminuria (3–30 mg/mmol), and macroalbuminuria (> 30 mg/mmol). Systolic blood pressure was divided into three ranges: <130 mm Hg, 131–139 mm Hg, and > 140 mm Hg.

The pharmacotherapies for blood pressure, diabetes and cholesterol were retrieved and the ATC codes are specified in Supplementary Table 1. The total number of days under care, hospitalizations, outpatient care visits, primary care visits, and emergency department visits were documented for each patient. Outpatient care and primary care visits were further categorized based on whether they involved consultations with a physician, nurse, or paramedical personnel. The study also recorded the number of follow-up visits in outpatient care, primary care, the emergency department, and the possibility of subsequent readmissions.

Visits to the emergency department, hospitalization and the date of death were registered. Number of days, until the first event occurred, was also registered. The Individual visits to hospital outpatient care or primary care visit to a physician or nurse within 30 days were registered.

### Statistical analysis

Descriptive statistics was used to describe characteristics of the study population, including age, sex, comorbidities, laboratory tests and medication. Continuous variables were presented with mean and Standard Deviation (SD) and categorical variables were presented with frequency and percentages. The comparison of continuous variables was performed using Student’s t-test and when several groups were compared, the ANOVA analysis was used. All statistical tests were 2-sided, unless otherwise specified, and *p* < 0.05 was used to identify significant differences.

Relative risks for an emergency department visit/hospitalization and mortality were estimated by Hazard ratios (HRs) with 95% confidence intervals (CI), which were calculated in two Cox regression analyses with adjustments made for age, sex, atherosclerotic CVD, glucose regulation, lipid levels, kidney function, blood pressure levels and pharmacotherapy for diabetes, blood pressure and cholesterol. Two Kaplan-Meier plots for the outcome of emergency department visits/hospitalizations and for the mortality were performed. Statistical analyses were performed in IBM SPSS Statistics 29.

### Ethical considerations

This study received ethical approval from the Swedish Ethical Review Board, at the Gothenburg Department of Medicine, under registration number 2020–05769. In this retrospective observational cohort study, the requirement for informed consent was waived, as it complied with the approvals granted by the Swedish Ethical Review Board. The methods and procedures employed in this research adhered to the relevant research guidelines and regulations.

## Results

A total of 1759 individuals with T2D who had a new CVD diagnosis were included in the current study. Out of these cases, 1173 (67%) individuals received their CVD diagnosis during a hospitalization, while 272 (16%) individuals were diagnosed in an outpatient care hospital setting, and 255 (14%) individuals received their diagnosis in primary care. Within the study cohort, 59 (3%) individuals were found to have been diagnosed in the emergency department.

The distribution of the specific new CVD diagnoses that included the individuals in the study included IHD in 1113 individuals (63%), ischemic cerebrovascular infarction in 581 individuals (33%), and peripheral artery disease in 151 individuals (9%), whereas some patients could have had more than one CVD diagnosis.

The main baseline characteristics are presented in Table 1 in the total cohort and their distribution of in-patient care, out-patient care, primary care, and emergency department according to the origin of diagnosis.

**Table 1:** Baseline characteristics, by the healthcare facilities responsible for first diagnosing the cardiovascular disease according to ICD-code

	In-patient care	Out-patient care	Primary care	Emergency department	Total	P-value
Total cohort	1173	272	255	59	1759	
*Age*						
Age, mean (SD)	75.2 (10.6)	73.2 (9.5)	75.2 (9.4)	74.2 (10.1)	74.9 (10.3)	0.02
*Sex*						
Women, n (%)	441 (38)	96 (35)	80 (31)	29 (49)	646 (37)	0.05
Men, n (%)	732 (62)	176 (65)	175 (69)	30 (51)	1113 (63)	
*Glucose levels*						
P-Glucose mmol/l, mean (SD)	9.5 (4.3)	8.8 (3.6)	8 (3.2)	8.6 (3.6)	9.2 (4.1)	< 0.001
P-Glucose < 8.5 mmol/mol	476 (48)	113 (53)	140 (66)	22 (60)	751 (52)	< 0.001
Missing P-Glucose	187 (16)	58 (21)	44 (17)	22 (37)	311 (18)	< 0.001
HbA1c, mean (SD)	57.3 (15.8)	57.1 (14.5)	53.4 (12.0)	53.1 (9.9)	56.4 (14.9)	0.01
*HbA1c levels*						
<52 mmol/mol, n (%)	284 (42)	66 (37)	102 (53)	15 (54)	467 (44)	0.049
52–70 mmol/mol, n (%)	286 (43)	85 (48)	74 (38)	10 (36)	455 (43)	
>70 mmol/mol, n (%)	101 (15)	28 (16)	17 (9)	3 (11)	149 (14)	
Missing, n (%)	502 (43)	93 (34)	62 (24)	31 (53)	688 (39)	
*Cholesterol-levels*						
Total-Cholesterol mmol/mol, mean (SD)	4.6 (1.2)	4.5 (1.1)	4.2 (1.1)	4.8 (1.2)	4.5 (1.2)	< 0.001
Cholesterol < 4.5, n (%)	387 (48)	123 (55)	154 (64)	15 (38)	679 (52)	
LDL-Cholesterol mmol/mol, mean (SD)	2.8 (2.1)	2.6 (1.0)	2.5 (1.0)	3 (1.0)	2.7 (1.0)	< 0.001
LDL-Cholesterol < 2.5 mmol/mol, n (%)	345 (43)	108 (49)	134 (57)	14 (35)	601 (46)	< 0.001
HDL-Cholesterol mmol/mol, mean (SD)	1.3 (0.4)	1.2 (0.4)	1.2 (0.3)	1.2 (0.3)	1.2 (0.4)	0.23
HDL-Cholesterol > 1.2 mmol/mol, n (%)	436 (56)	112 (52)	126 (57)	17 (44)	691 (55)	0.33
Triglycerides mmol/mol, mean (SD)	2 (1.3)	2.3 (1.7)	1.8 (0.9)	2.3 (1.3)	2.1 (1.3)	0.002
*Renal function*						
eGFR, mean (SD)	58.7 (20.8)	62.8 (19.4)	64 (15.7)	57.2 (18)	59.9 (20)	< 0.001
eGFR > 60 ml/min, n (%)	574 (53)	148 (58)	138 (62)	53 (56)	913 (55)	0.01
eGFR 30–60 ml/min, n (%)	392 (36)	86 (34)	78 (35)	33 (35)	589 (36)	
eGFR < 30 ml/min, n (%)	119 (11)	21 (8)	7 (3)	9 (10)	156 (9)	
Troponin T, mean (SD)	299.5 (1007.7)	157.1 (752.8)	20.1 (21.7)	73.4 (191.8)	262.8 (941.2)	0.04
Number of Troponin T, n (%)	889 (76)	69 (25)	67 (26)	48 (81)	1073 (61)	
*Clinical findings*						
Heart rate, mean (SD)	76.3 (17.4)	76.1 (17.0)	72.4 (13.2)	77.5 (15.7	75.8 (16.8)	0.01
*Blood pressure*						
Systolic mm Hg, mean (SD)	133.9 (20.3)	1325 (19.5)	136.7 (18.3)	134.3 (21.4)	134.1 (20)	0.11
Diastolic mm Hg, mean (SD)	75.4 (11.2)	73.9 (10.5)	75.8 (10.3)	73.8 (12.0)	75.2 (11.0)	0.13

*Note* n = number, SD = standard deviation, HbA1c = hemoglobin A1c, HDL-cholesterol = high-density lipoprotein cholesterol, LDL-cholesterol = Low-density lipoprotein cholesterol, eGFR = estimated glomerulofiltration rate

In total, there were 1341 (76%) individuals in the study that survived during the one-year follow-up period. The number of individuals having follow-up of HbA1c at the end of the study was 1227 (91% of the survivors) individuals and for eGFR, it was 1224 (91% of the survivors). Metformin was the predominant choice of diabetes medication, with 1112 individuals (63%) prescribed this treatment. Treatment of renin-angiotensin-aldosterone system inhibitors (RAASi) occurred in 1210 (69%) individuals and beta blockers in (64%) 1133 and 1378 (78%) individuals received statin therapy. Table 2 provides a summary of the laboratory and blood pressure follow-up as well as the pharmacotherapy prescribed starting from the index date and continuing throughout the study period. The average number of days hospitalized during the study period was 6.5 days for the study cohort. The total number of events related to emergency department visits and hospitalizations amounted to 2171, with the distribution being 1839 (85%) of these events occurring within the in-patient care group, 179 in out-patient care, 85 (4%) in primary care, and 68 events (3%) in the emergency department group. The number of follow-up visits after first being diagnosed with CVD was 1.1 for out-patient care in the hospital setting and 10.1 for visits to primary care. There were 900 (51%) of the individuals having a follow-up visit to primary care ≤ 30 days after index of CVD diagnosis and the corresponding number for hospital out-patient care was 146 (8%). Healthcare utilization is displayed in Table 2.

**Table 2:** Illustration of the course of follow-up during the study period regarding mortality, laboratory findings, blood pressure, pharmacotherapy and healthcare utilization

	In-patient care	Out-patient care	Primary care	Emergency department	Total	p-value
Total cohort	1173	272	255	59	1759	
Deceased, n (%)	340 (29)	40 (15)	19 (8)	19 (32)	418 (24)	< 0.001
*Laboratory findings*						
*HbA1c levels*						
< 52 mmol/mol, n (%)	390 (52)	114 (53)	145 (57)	36 (62)	654 (53)	0.40
52–70 mmol/mol, n (%)	268 (36)	82 (38)	79 (35)	17 (29)	437 (36)	
> 70 mmol/mol, n (%)	94 (13)	20 (9)	17 (8)	5 (9)	136 (11)	
LDL-cholesterol < 2.5, n (%)	532 (45)	129 (66)	91 (66)	31 (54)	783 (70)	0.01
eGFR > 60 ml/min, n (%)	410 (35)	95 (54)	75 (56)	34 (47)	614 (50)	0.24
eGFR 30–60 ml/min, n (%)	329 (28)	68 (39)	49 (37)	30 (42)	476 (39)	
eGFR < 30 ml/min, n (%)	104 (9)	12 (7)	10 (8)	8 (11)	134 (11)	
*Blood pressure*						
< 100 mm Hg, n (%)	11 (1)	9 (4)	1 (< 1)	3 (4)	24 (18)	0.006
100–130 mm Hg, n (%)	300 (37)	71 (31)	63 (29)	20 (30)	454 (34)	
> 130 mm Hg, n (%)	506 (62)	148 (65)	154 (71)	43 (65)	851 (64)	
*Pharmacotherapy*						
Metformin, n (%)	684 (61)	206 (70)	177 (69)	45 (47)	1112 (63)	< 0.001
Sulfonylurea, n (%)	41 (4)	20 (7)	12 (5)	2 (2)	75 (5)	0.08
GLP1, n (%)	122 (11)	43 (15)	28 (11)	15 (16)	208 (12)	0.21
DPP4, n (%)	199 (18)	46 (16)	49 (19)	17 (18)	311 (18)	0.69
SGLT2, n (%)	133 (12)	44 (15)	39 (15)	6 (6)	222 (13)	0.07
Insulins, n (%)	306 (28)	91 (31)	35 (14)	20 (21)	452 (26)	< 0.001
Dietary diabetes treatment, n (%)	223 (20)	43 (15)	49 (19)	27 (28)	342 (19)	0.03
RAASi, n (%)	766 (69)	211 (72)	177 (69)	56 (58)	1210 (69)	0.10
Calcium antagonists, n (%)	424 (38)	116 (40)	112 (44)	32 (33)	684 (39)	0.23
Beta blockers, n (%)	717 (64)	196 (67)	160 (63)	60 (63)	1133 (64)	0.77
Diuretics (any of below), n (%)	465 (42)	124 (42)	58 (23)	36 (38)	683 (40)	< 0.001
Aldosterone antagonists, n (%)	175 (16)	45 (15)	15 (6)	14 (15)	249 (14)	< 0.001
Furosemide, n (%)	381 (34)	102 (35)	45 (18)	26 (27)	554 (32)	< 0.001
Thiazide, n (%)	61 (6)	15 (5)	14 (6)	5 (5)	95 (5)	1.00
Statin, n (%)	875 (78)	232 (79)	214 (84)	57 (59)	1378 (78)	< 0.001
Ezetimibe, n (%)	53 (5)	14 (5)	13 (5)	6 (6)	86 (5)	0.93
*Healthcare utilization*						
*Hospital care setting*						
In-patient care						
Hospital admissions, mean (SD)	1.5 (1.5)	0.6 (1.1)	0.3 (0.8)	1.1 (1.2)	1.2 (1.4)	< 0.001
In-patient care days, mean (SD)	8.5 (11.7)	3.1 (7.4)	1.3 (4.9)	5.4 (9.6)	6.5 (10.7)	< 0.001
Out-patient care						
Physician visits, mean (SD)	0.5 (3.1)	1.2 (12.9)	0.2 (1.3)	0.8 (1.3)	0.6 (1.1)	< 0.001
Nurse visits, mean (SD)	0.5 (0.5)	1.1 (1.1)	0.3 (1.3)	0.2 (0.4)	0.5 (5.7)	0.33
*Primary care setting*						
Primary care visits, mean (SD)	10.1 (12.5)	11.1 (11.7)	9.1 (9.1)	9.8 (12.5)	10.1 (11.9)	0.28
Physician visits, mean (SD)	3.5 (4.3)	3.2 (3.5)	3.6 (3.5)	3.6 (3.7)	3.5 (4.0)	0.77
Nurse visits, mean (SD)	6.6 (10.2)	7.9 (9.6)	5.5 (6.9)	6.2 (9.8)	6.6 (9.8)	0.049
*Out-patient care revisits* *≤* *30 days*						
Hospital out-patient care, n (%)	91 (8)	33 (11)	10 (4)	12 (12)	146 (8)	0.007
Primary care, n (%)	497 (45)	133 (45)	222 (87)	48 (50)	900 (51)	< 0.001

*Note* n = number, SD = standard deviation, HbA1c = hemoglobin A1c, LDL-cholesterol = low-density lipoprotein cholesterol, eGFR = estimated glomerulofiltration rate, GLP1 = Glucagon-like peptide-1 agonists, DPP4 = dipeptidyl peptidase 4 inhibitors, SGLT-2 = sodium-glucose cotransporter-2 inhibitors, RAASi = Renin-angiotensin-aldosterone system inhibitors, Diuretics = aldosterone antagonists or furosemide or thiazide

The overall all-cause mortality amounted to 418 (24%) individuals within one year. This was comprised of 19 (4.5%) individuals of the deceased who died with an initial diagnosis made in the emergency department, 19 (4.5%) individuals whose CVD diagnosis originated in primary care, and 40 (10%) individuals from the hospital outpatient care group. There were 340 (81%) individuals from the hospital inpatient care group. Mortality rates for individuals receiving primary care and hospital outpatient care exhibited a more gradual increase, evenly distributed over one year. Figure 1 depicts the evolution of mortality as well as events occurring during emergency department visits/hospitalizations throughout the study period of one year from index.

Cox regression analysis was utilized to study hospital admissions/visits to the emergency department adjusted for first encounter of the CVD diagnosis, sex, age, pharmacotherapies, and ≤ 30 days follow-up in both hospital outpatient settings and primary care (Table 3). Individuals who received their diagnosis in the emergency department exhibited an elevated relative risk as well as individuals with higher age. Treatment with statins and RAASi, visits to both hospital outpatient care and primary care, contributed to a decreased relative risk.

**Table 3:** Cox regression analysis to assess hospital admissions/emergency department visits

		Hazard	95.0% CI for HR		p-value
		ratio	Lower	Upper	
*Diagnose origin*					
IPC	Reference	1.00			
OPC		0.89	0.68	1.16	0.06
Primary care		0.52	0.35	0.77	< 0.001
Emergency department		1.23	0.78	1.94	0.80
*Sex and age*					
Women		0.88	0.72	1.07	0.20
Age		1.02	1.00	1.03	0.01
*Systolic blood pressure*					
< 100 mm Hg	Reference	1.00			
100–130 mmHg		0.61	0.32	1.14	0.31
> 130 mm Hg		0.54	0.29	1.00	0.21
*Renal function*					
eGFR > 60 ml/min	Reference	1.00			
eGFR 30–60 ml/min		0.87	0.69	1.09	0.30
eGFR < 30 ml/min		0.85	0.58	1.24	0.40
*Glucose regulation*					
HbA1c < 52 mmol/ml	Reference	1.00			
HbA1c 52–70 mmol/ml		0.85	0.69	1.05	0.43
HbA1c > 70 mmol/ml		0.76	0.54	1.07	0.90
LDL-cholesterol ≥ 2.5 mmol/ml		1.00			
LDL-cholesterol < 2.5 mmol/ml		1.05	0.84	1.32	0.67
*Pharmacotherapy*					
Insulin		1.29	1.04	1.61	0.02
Metformin		0.88	0.70	1.10	0.25
DPP4		1.38	1.10	1.74	0.006
GLP1		1.04	0.78	1.38	0.81
SGLT2		1.01	0.77	1.34	0.93
Aldosterone antagonist		1.63	1.27	2.08	< 0.001
Betablocker		1.34	1.08	1.67	0.008
Calcium blocker		1.21	0.99	1.47	0.06
RAASi		1.01	0.80	1.26	0.95
Statin		0.97	0.71	1.32	0.82
*Healthcare utilization*					
Hospital out-patient care visit > 30 days	Reference	1.00			
Hospital out-patient care visit ≤ 30 days		2.19	1.70	2.82	< 0.001
Primary care visit > 30 days	Reference	1.00			
Primary care visit ≤ 30 days		0.86	0.70	1.05	0.13

*Note* CI = confidence interval, IPC = in-patient care (hospitalization), OPC = out-patient care in hospital setting, HbA1c = hemoglobin A1c, LDL-cholesterol = Low-density lipoprotein cholesterol, eGFR = estimated glomerulofiltration rate, GLP1 = Glucagon-like peptide-1 agonists, DPP4 = dipeptidyl peptidase 4 inhibitors, SGLT-2 = sodium-glucose cotransporter-2 inhibitors, RAASi = Renin-angiotensin-aldosterone system inhibitors

A second Cox regression analysis to study mortality was adjusted for age, systolic blood pressure levels, blood glucose levels, renal function values, and treatment interventions is displayed in Table 4.

**Table 4:** Cox regression analysis to assess mortality

		Hazard	95.0% CI for Exp(B)		
		ratio	Lower	Upper	p-value
*Origin of diagnosis*					
IPC	Reference	1.00			
OPC		0.37	0.20	0.67	< 0.001
Primary care		0.34	0.18	0.66	< 0.001
Emergency department		0.56	0.25	1.26	0.16
*Sex and age*					
Women		1.08	0.75	1.56	0.67
Age		1.05	1.03	1.08	< 0.001
*Systolic blood pressure*					
< 100 mmHg	Reference	1.00			
100–130 mmHg		0.38	0.19	0.77	0.02
> 130 mmHg		0.26	0.13	0.52	< 0.001
*Glucose regulation*					
P-Glucose > 8,5 mmol/ml	Reference	1.00			
P-Glucose ≤ 8,5 mmol/ml		0.91	0.62	1.31	0.60
HbA1c < 52 mmol/mol	Reference	1.00			
HbA1c 52–70 mmol/mol		1.49	1.07	2.56	0.02
HbA1c > 70 mmol/mol		1.46	1.18	3.82	0.01
*Kidney function*					
eGFR > 60 ml/min	Reference	1.00			
eGFR 30–59 ml/min		1.49	0.86	2.56	0.36
eGFR < 30 ml/min		1.49	0.86	2.59	0.15
*LDL-Cholesterol*					
LDL < 2,5 mmol/ml	Reference	1.00			
LDL ≥ 2.5 mmol/ml		1.46	1.01	2.11	0.04
*Pharmacotherapy*					
Insulin		0.80	0.52	1.22	0.30
Metformin		0.41	0.28	0.62	< 0.001
DPP4		0.85	0.55	1.32	0.47
GLP1		0.96	0.54	1.73	0.90
SGLT2		0.20	0.06	0.64	0.01
Aldosterone antagonist		0.80	0.52	1.24	0.32
Betablocker		0.97	0.66	1.44	0.89
Calcium antagonist		0.70	0.48	1.01	0.06
RAASi		0.74	0.51	1.07	0.11
Statin		0.39	0.27	0.57	< 0.001
*Follow-up visit*					
Hospital outpatient care > 30 days	Reference	1.00			
Hospital outpatient care ≤ 30 days		0.79	0.38	1.64	0.53
Primary care > 30 days	Reference	1.00			
Primary care ≤ 30 days		0.53	0.37	0.77	< 0.001

*Note* CI = confidence interval, IPC = in-patient care (hospitalization), OPC = out-patient care in hospital setting, HbA1c = hemoglobin A1c, LDL-cholesterol = Low-density lipoprotein cholesterol, eGFR = estimated glomerulofiltration rate, GLP1 = Glucagon-like peptide-1 agonists, DPP4 = dipeptidyl peptidase 4 inhibitors, SGLT-2 = sodium-glucose cotransporter-2 inhibitors, RAASi = Renin-angiotensin-aldosterone system inhibitors

The HR for outpatient care and primary care encountering the CVD diagnosis is associated with lower mortality risk. Pharmacotherapy with metformin, SGLT-2 statins, increased systolic blood pressure and a follow-up visit in primary care ≤ 30 days was associated with a lowered risk for mortality. There was an association of increased risk for mortality regarding advanced age.

## Discussion

In this retrospective study of individuals with T2D and a new CVD diagnosis, the majority of T2D individuals initially received their new CVD diagnosis during inpatient care. Subsequently, the follow-up was primarily conducted in primary care. The overall mortality rate in the study group was 24%, with the highest mortality rate observed among those diagnosed either during inpatient care or in the emergency department. Factors such as diagnosis in the emergency department, advanced age, and visits < 30 days to outpatient care were associated with an increased risk of mortality. However, the study found that treatment with statins was associated with a lowered risk for both emergency department visits/hospitalization and mortality. Blood pressure higher than 100 mm Hg was associated with a lowered risk for mortality.

Lowering HbA1c levels, particularly achieving near-normal levels (HbA1c < 53 mmol/mol), is associated with reduced microvascular complications [23, 24]. However, the impact on macrovascular disease is complex [25]. In our study, 47% of individuals maintained HbA1c levels above 52 mmol/mol during follow-up, indicating inadequate glucose control, which did not affect emergency department visits or hospitalization. This aligns with the definition of CVD events as macrovascular [26]. Nevertheless, it had a more detrimental effect on retinopathy and diabetic nephropathy risks. The 10-year follow-up of the UKPDS post-trial study revealed that the earlier period of tight blood pressure control did not show sustained benefits in terms of macrovascular events, mortality, or microvascular complications [26]. In our one-year study, the impact of blood pressure control on cardiovascular events was aligned with expectations. Importantly, low blood pressure emerges as a marker associated with an adverse prognosis for mortality. Prior studies have established an association between elevated LDL-cholesterol levels and increased rates of cardiovascular events and mortality [27, 28]. Our study indicates an association between high LDL-cholesterol and increased mortality risk, although the evidence is less conclusive regarding the risk of a cardiovascular event, possibly due to the study’s relatively short duration in this context. In this investigation, there was an association between enhanced mortality risk and the monitoring and regulation of HbA1c, LDL-cholesterol, and renal impairment. Remarkably, good control of these variables has been achieved to a notable extent, considering the 24% mortality rate observed among individuals over the study duration. Individuals subjected to treatment with metformin, SGLT-2 inhibitors, and statins exhibit a diminished mortality risk. However, establishing a similar effect concerning the risk of hospitalization or emergency department event was lacking.

Prior research has indicated that the combination of T2D and CVD indicates a higher risk of hospitalization [5, 6]. Our study supports these findings, demonstrating an average hospital stay of 6.5 days following discharge. In contrast to medical conditions that necessitate frequent hospitalization, such as heart failure, the average duration of hospitalization for heart failure is 6.6 days [19]. Regarding the follow-up visits after the onset of CVD diagnosis, our study found that the frequency of visits to the hospital’s outpatient care is relatively low, even though most cases of newly diagnosed CVD are identified during inpatient care. The primary mode of follow-up care predominantly occurs in primary care settings, which can be attributed to the common practice of monitoring T2D individuals in primary care. Our study revealed that individuals diagnosed in the emergency department faced an elevated risk of hospitalization and emergency department visits during the study period. Moreover, increased age and early re-visits to hospital outpatient care after being diagnosed with CVD were associated with a higher risk of experiencing serious adverse events. The likelihood of early re-visits to the hospital’s outpatient department leading to increased hospitalization or emergency department visits may be attributed to the severity of CVD in these individuals.

Previous studies have indicated an elevated mortality rate in individuals with T2D and CVD compared to the control group [4, 7]. This current study also affirms an elevated mortality rate of 24% in individuals with both T2D and CVD. This mortality rate is notably higher than the 17% reported in a prior study, with the disparity likely attributed to the inclusion of individuals at the onset of CVD in the present study. The study’s findings emphasize that mortality rates were notably elevated, especially within the initial two weeks and among individuals diagnosed in the emergency department. A similar pattern with a high mortality rate was previously reported in a population of patients with heart failure [20]. This underscores the substantial CVD risks associated with T2D and the importance of precise and relatively intensive monitoring of these individuals. Factors associated with increased mortality are increasing age and high cholesterol levels, while in this study it was important where the patient received the CVD diagnosis. In contrast, treatment with metformin, SGLT-2 antagonists and statins is associated with reduced mortality risk. Likewise, follow-up in primary care within 31 days after CVD diagnosis was established with an associated reduced risk of mortality. Consequently, prioritizing the follow-up of T2D individuals in primary care in Sweden is warranted, and it is reasonable to expect that primary care bears a responsibility for monitoring critical risk factors.

### Strengths and limitations

This study has the following strengths: large sample size, data were based on RHIP which has good quality and complete information on the medications used and biomarkers. However, there are some limitations. It has been possible to examine the duration of diabetes, but since the lookback period only extended to 2011, this variable was not assessed to be reliable. It is likely that individuals with T2D have had the diagnosis for a significantly longer time, which would significantly affect the duration times. There is a lack of information on other potential confounding factors, for example, socioeconomic status. The study encompassed a total of 1759 participants, a figure that may be considered modest in size in certain contexts. Additionally, the study used Cox regression analysis with a substantial number of variables - a factor that could be viewed as a limitation.

In the study, the need for cardiology interventions was not recorded or investigated, which is a factor that should affect the need for readmission, visits to the emergency department and follow-up. As reliable data have not been able to be compiled, this is not included in the analyses. Given that this was a retrospective population-based study, it was not feasible to arrive at causal conclusions. Future prospective studies are needed to make causal conclusions about the observed associations.

## Conclusion

The majority of these individuals tend to receive their CVD diagnosis during hospital inpatient care and the follow-up after being diagnosed with CVD is mainly in primary care. Factors associated with an elevated risk of serious adverse events during the follow-up period primarily include advancing age, although this risk is mitigated in individuals receiving statin treatment. Aging and low blood pressure are also linked to an increased risk of mortality, but conversely, pharmacotherapy involving SGLT-2, metformin, statins, and early follow-up in outpatient primary care is associated with reduced risk. As a result, primary care plays a crucial role in the follow-up process, significantly raising the expectations for primary care to monitor risk factors and ensure these patients receive recommended pharmacotherapy.

### Electronic supplementary material

Below is the link to the electronic supplementary material.

Supplementary Material 1

Supplementary Material 2

## Data Availability

The datasets generated and analyzed in the current study are not publicly available due to the Swedish Health and Medical Services Act’s regarding the Secrecy Act but could be available upon a reasonable request made to the corresponding author and followed by a specific review by the Regional Consultative Committee for data collection in Region Halland.

## Abbreviations

CI: confidence interval
CVD: cardiovascular disease
Diuretics: aldosterone antagonists or furosemide or thiazide
DPP4: dipeptidyl peptidase 4 inhibitors
eGFR: estimated glomerular filtration rate
GLP1: Glucagon-like peptide-1 agonists HbA1c = glycated hemoglobin
HDL-cholesterol: high-density lipoprotein cholesterol
HR: Hazard ratio
IPC: in-patient care (hospitalization)
LDL-cholesterol: low-density-lipoprotein cholesterol
n: number
OPC: out-patient care in hospital setting
RAASi: renin-angiotensin-aldosterone-system inhibition (includes Angiotensin-converting-enzyme inhibitors, Angiotensin receptor blockers and Angiotensin receptor neprilysin)
RHIP: regional healthcare information platform
SD: standard deviation
SGLT-2 inhibitors: sodium-glucose cotransporter-2 inhibitors
T2D: type 2 diabetes
