# Supplementary material for: Factors influencing hospitalization or emergency department visits and mortality in type 2 diabetes following the onset of new cardiovascular diagnoses in a population-based study
Source: Cardiovasc Diabetol. 2024 Apr 10;23:124. doi: 10.1186/s12933-024-02211-4 (PMC11007935; doi:10.1186/s12933-024-02211-4)
Supplement: Supplementary file 1 — Supplementary Material 1 [file 12933_2024_2211_MOESM1_ESM.docx]

Appendix Figure 1. Displays the study procedure.


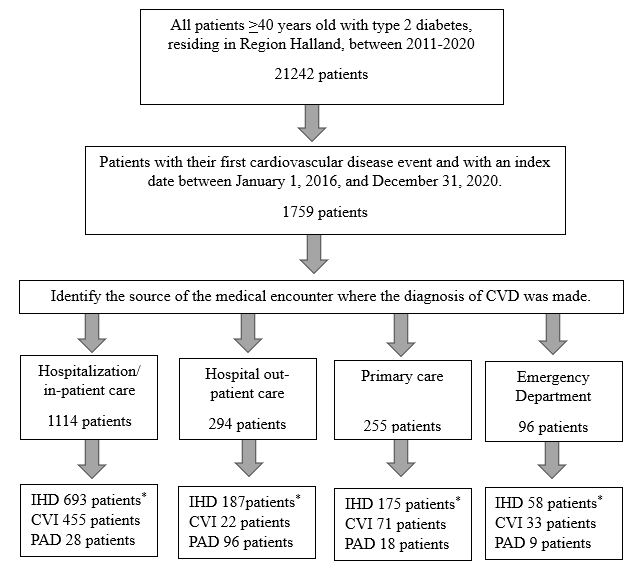


Note; IHD=ischemic heart disease, CVI=cerebrovascular insult, PAD=peripheral artery disease (ICD-codes displayed in Appendix Table 1).

^*^ A patient could receive multiple cardiovascular diagnoses on the index date.
