# Supplementary material for: Factors influencing hospitalization or emergency department visits and mortality in type 2 diabetes following the onset of new cardiovascular diagnoses in a population-based study
Source: Cardiovasc Diabetol. 2024 Apr 10;23:124. doi: 10.1186/s12933-024-02211-4 (PMC11007935; doi:10.1186/s12933-024-02211-4)
Supplement: Supplementary file 2 — Supplementary Material 2 [file 12933_2024_2211_MOESM2_ESM.docx]

**Supplementary Tables**

Appendix Table 1. Table of diagnosis and ICD-10 codes for each disease.

| **Disease** | **ICD-10** |
| --- | --- |
| Diabetes (DT2) | E11, E13, E14 |
| Cardiovascular disease |  |
| Ischemic heart disease (IHD) | I20-I25 |
| Acute myocardial infarction (previous) (AMI) | I21-I22, I25.2, I25.6 |
| Cerebrovascular insult (CVI) | I63, I672, I678, I679, I693, G45 |
| Ischemic | I63, I672, I678, I679, I693 |
| Transitory ischemic attack (TIA) | G45 |
| Peripheral artery disease (PAD) | I70.2, I73.9, I74.2-9 |
| Heart failure (HF) | I50, I11.0, I13.0, I13.2, I42 |
| Chronic kidney disease (CKD) | N17-N19, I12.0-I12.9, I13.1, I13.2, N00-N08, N10-N16 N08.3, E10.2, E11.2, E12.2, E13.2, E14.2, Z49, Z99.2 |
| Atrial fibrillation (AF) | I48 |
| Chronic obstructive pulmonary disease (COPD) | J44 |

Appendix Table 2. Table of pharmacotherapies and corresponding ATC-codes.

| **Drug** | **ATC code** | |
| --- | --- | --- |
|  | |  |
| Low dose acetylic salicylic acid  (ASA) | | B01AC06 |
| Statins | | C10AA |
| Angiotensin-converting enzyme inhibitors (ACEi) | | C09A, C09B |
| Angiotensin receptor blockers (ARB) | | C09C, C09D (exclude C09DX04) |
| Betablockers | C07 | |
| Calcium antagonists | C08D | |
| Diuretics     Loop-diuretics     Thiazides | C03  C03C  C03A | |
| Aldosterone antagonists | C03DA | |
| Angiotensinreceptor neprilysin inhibitors (ARNI) | C09DX04 | |
| Digitalis | C01AA05 | |
| Warfarin/Coumarin | B01AA | |
| Receptor P2Y12 antagonists | B01AC04, B01AC22, B01AC24 | |
| Other antiplatelets | B01AC07, B01AC09, B01AC11,  B01AC13, B01AC16, B01AC17, B01AC21 | |
| Nitrates | C01DA | |
| Sodium-glucose cotransporter-2 inhibitors (SGLT-2) | A10BK01, A10BK02, A10BK03 or A10BD15, A10BD16, A10BD20 (in combination) | |
| Metformin | A10BA, or A10BD (in combination) | |
| Sulfonylurea (SU) | A10BB | |
| Dipeptidyl peptidase 4 inhibitors  (DPP-4) | A10BH, or A10BD07, A10BD08, A10BD10 (in combination) | |
| Glucagon-like peptide-1 receptor agonist (GLP-1RA) | A10BJ01, A10BJ02, A10BJ03, A10BJ05, A10BJ06 | |
| Metiglinides | A10BX02, A10BX03 or A10BD03, A10BD04, A10BD05 (in combination) | |
| Glitazone | A10BG | |
| Acarbose | A10BF | |
| *Insulins* |  | |
| Short-acting insulins | A10AB | |
| Intermediate-acting (isophane) insulins | A10AC | |
| Premixed insulin  insulins | A10AD | |
| Long-acting insulins | A10AE | |
